# The role of cutaneous manifestations in the diagnosis of the Ehlers‐Danlos syndromes

**DOI:** 10.1002/ski2.140

**Published:** 2022-07-15

**Authors:** Natasha Stembridge, Brent J. Doolan, Mark E. Lavallee, Ingrid Hausser, F. Michael Pope, Suranjith L. Seneviratne, Ingrid M. Winship, Nigel P. Burrows

**Affiliations:** ^1^ Department of Dermatology Cambridge University Hospitals NHS Foundation Trust Cambridge UK; ^2^ St John's Institute of Dermatology School of Basic and Medical Biosciences King's College London London UK; ^3^ Guy's and St Thomas' NHS Foundation Trust London UK; ^4^ Department of Orthopedics University of Pittsburgh Medical Center of Central PA Pittsburgh Pennsylvania USA; ^5^ Institute of Pathology Heidelberg University Hospital Heidelberg Germany; ^6^ Department of Dermatology Chelsea and Westminster Hospital NHS Foundation Trust (West Middlesex University Hospital) London UK; ^7^ Institute of Immunity and Transplantation Royal Free Hospital and University College London London UK; ^8^ Nawaloka Hospital Research and Education Foundation Nawaloka Hospitals Colombo Sri Lanka; ^9^ Department of Genetic Medicine The Royal Melbourne Hospital Melbourne Victoria Australia; ^10^ Department of Medicine The University of Melbourne Melbourne Victoria Australia

## Abstract

The Ehlers‐Danlos syndromes (EDS) comprise a group of inherited connective tissue disorders presenting with features of skin hyperextensibility, joint hypermobility, abnormal scarring and fragility of skin, blood vessels and some organs. The disease is generally diagnosed through the cluster of clinical features, though the addition of genetic analysis is the gold standard for diagnosis of most subtypes. All subtypes display skin manifestations, which are essential to the accurate clinical diagnosis of the condition. Furthermore, cutaneous features can be the first and/or only presenting feature in some cases of EDS and thus understanding these signs is vital for diagnosis. This review focuses on particular cutaneous features of each EDS subtype and their clinical importance. Provision of a specific diagnosis is important for management, prognosis and genetic counselling, often for family members beyond the individual.

## Graphical abstract

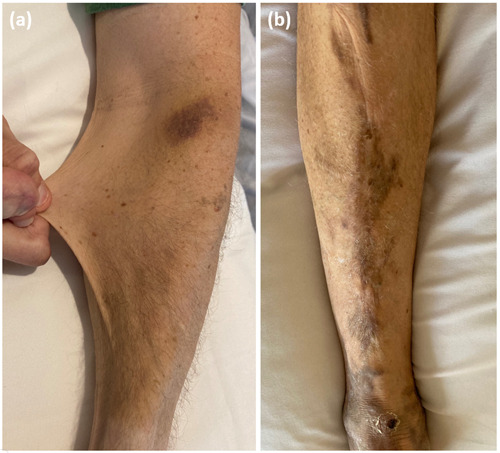

1

**What is already known about this topic?**

- The Ehlers‐Danlos syndromes (EDS) are a group of inherited connective tissue disorders with variable cutaneous fragility, joint hypermobility and systemic manifestations.
- There are currently 14 proposed sub‐types, all of which display skin features within the minor and/or major criteria for diagnosis of Ehlers‐Danlos syndrome.

**What does this study add?**

- This review focuses on the main cutaneous findings of the Ehlers‐Danlos syndrome subtypes and provides clinicians with a helpful guide to the assessment of these clinical features to aid in diagnosis.

## 2 INTRODUCTION

The Ehlers‐Danlos syndromes (EDS) are a group of inherited connective tissue disorders with variable cutaneous fragility, joint hypermobility and systemic manifestations.^1^ The term ‘Ehlers‐Danlos Syndrome’ encompasses a group of 13 subtypes with marked clinical and genetic heterogeneity arising from defects in fibrillar collagens and other extracellular matrix proteins.^2^ Following the latest 2017 EDS international classification, one additional subtype has been proposed, classical‐like type 2 EDS (clEDS2), caused by variants in the *AEBP1* gene.^3^, ^4^ All EDS subtypes exhibit significant cutaneous features within the major and/or minor clinical criteria (Table 1). Furthermore, cutaneous signs may sometimes be the first clinical feature and are a helpful characteristic in EDS subclassification and establishing an accurate diagnosis, prognosis and management. Thus, this review focuses on the skin manifestations in EDS to support clinicians in their clinical assessments.

**TABLE 1:** An outline of Ehlers‐Danlos syndrome subtypes, their associated genetic mutation and affected protein and most common cutaneous features (adapted from Malfait et al., 2017^3^)

Number	EDS subtype	Inheritance gene mutation	Affected protein	Skin features^a^
1	Classical	AD		*Hyperextensibility*
		Most *COL5A1* or *COL5A2*	Type V collagen	*Atrophic scarring*
		Rare *COL1A1*	Type I collagen	
2	Vascular	AD		Bruising without trauma and on unusual sites like cheeks and back
		Most *COL3A1*	Type III collagen	Thin translucent skin with increased venous visibility
		Rare *COL1A1*	Type I collagen	Acrogeria
3	Periodontal	AD		*Pretibial plaques*
		*C1R*	C1r	Easy bruising, skin hyperextensibility, wide or atrophic scarring.
		*C1S*	C1s	Acrogeria
4	Dermatosparaxis	AR		*Extreme skin fragility with congenital/post‐natal skin tears*.
		*ADAMTS2*	ADAMTS‐2	*Redundant almost lax skin with excessive folds at wrists and ankles*
				*Increased palmar wrinkling*
				*Severe bruisability with risk of subcutaneous haematomas and haemorrhage*
				Soft and doughy skin texture
				Skin hyperextensibility
				Atrophic scars
5	Cardiac‐valvular	AR	Type I collagen	*Hyperextensibility*
		*COLA1A2*		*Atrophic scars*
				*Thin skin*
				*Easy bruising*
6	Classical‐like	AR	Tenascin XB	Hyperextensibility with velvety skin texture
		*TNXB*		Absence of atrophic scarring
				Easy bruisable skin/spontaneous ecchymoses
7	Hypermobile	AD	Unknown	*Soft or velvety skin*
		Unknown		*Mild skin hyperextensibility*
				*Unexplained striae*
				*Atrophic scarring of at least two sites without papyraceous or haemosiderin scars Bilateral piezogenic papules*.
8	Arthrochalasia	AD	Type I collagen	*Skin hyperextensibility*
		*COL1A1*, *COL1A2*		Atrophic scars
				Easy bruising
9	Kyphoscoliotic	AR		Hyperextensibility
		*PLOD1*	LH1	Bruising
		*FKBP14*	FKBP22	Atrophic scarring (*PLOD1*)
				Follicular hyperkeratosis (*FKBP1*)
10	Brittle cornea syndrome	AR		Translucent skin.
		*ZNF469*	ZNF469	Soft, velvety skin.
		*PRDM5*	PRDM5	
11	Spondylodysplastic	AR		Hyperextensibility.
		*B4GALT7*	B4GalT7	Soft, doughy skin
		*B3GALT6*	B3GalT6	
		*SLC39A13*	ZIP13	
12	Musculocontractural	AR		*Hyperextensibility*.
		*CHST14*	D4ST1	*Easy bruising*.
		*DSE*	DSE	*Fragility with atrophic scars*.
				*Increased palmar wrinkling*.
13	Myopathic	AD or AR	Type XII collagen	Soft, doughy skin.
		*COL12A1*		Atrophic scars
14	Classical‐like Type‐2 (AEBP1‐related EDS)	AR	ECM‐associated adipocyte enhancer‐binding protein 1	Formal diagnostic not yet evaluated: Similarities with cEDS with skin hyperextensibility and atrophic scarring
		*AEBP1*		

*Note*: NB: Italicized skin features represent major clinical features.

Abbreviations: AD, Autosomal dominant; AR, Autosomal recessive; ECM, Extra‐cellular matrix; EDS, Ehlers‐Danlos syndrome.

^a^ Those listed in italics are part of the major diagnostic clinical criteria for that subtype.

## 3 CLASSICAL EHLERS DANLOS SYNDROME

Classical EDS (cEDS) usually has typical skin signs with some variations due to genetic penetrance and individual expression.^3^ Over 90% of cEDS cases are caused by heterozygous pathogenic variants in *COL5A1* or *COL5A2*, which dysregulate the collagen assembly of fibrillar collagens.^5^ A specific mutation (p.Arg312Cys) in *COL1A1* results in a phenotype of cEDS but with a propensity to vascular rupture.^6^ These mutations can cause an array of skin features, as well as mucocutaneous, ocular and facial features, which may assist in a clinical diagnosis.^7^

### 3.1 Skin hyperextensibility

**FIGURE 1:**
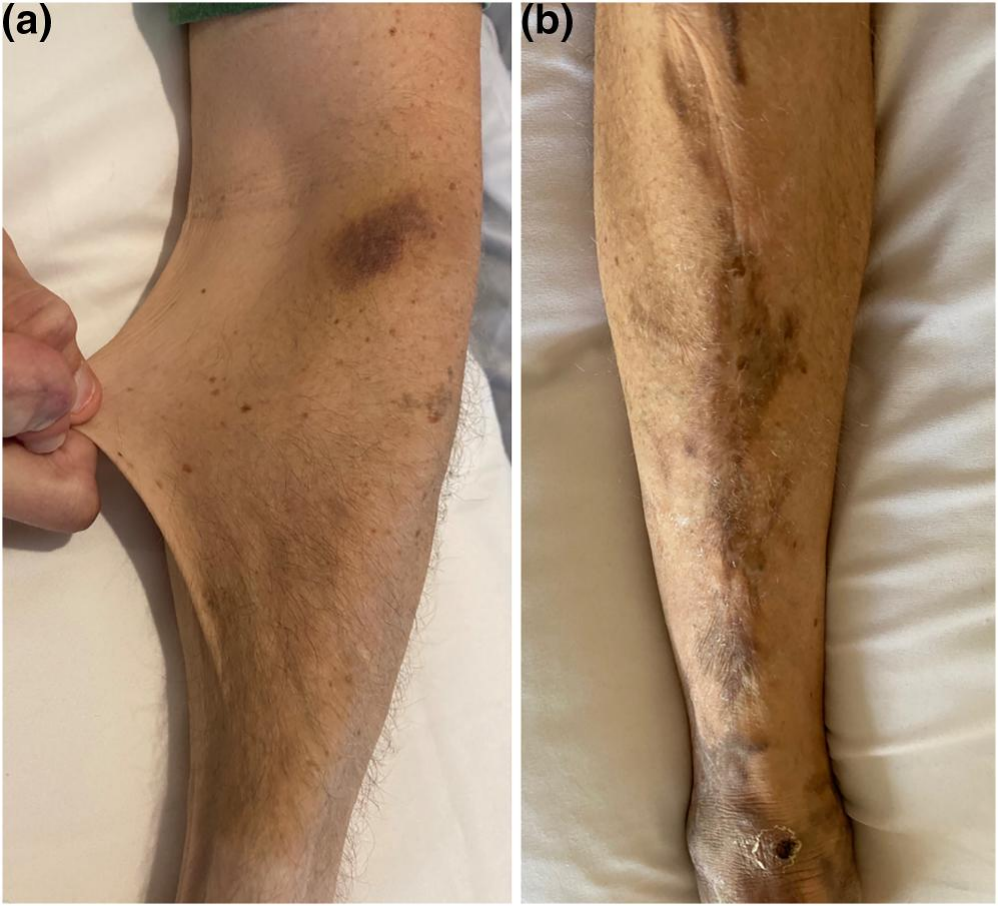
A patient with classical Ehlers‐Danlos syndrome, showing (a) hyperextensible skin and bruising and, (b) atrophic scarring with haemosiderin deposition on a bony prominence

This refers to the ability to stretch the skin beyond its normal range. Differentiation between the normal population, patients with hypermobile spectrum disorders and EDS using skin extensibility remains challenging, even for experts. In EDS, the elasticity of the skin is preserved, allowing the extended skin to recoil back to its original position.^1^ It has been suggested that there is a stress‐strain curve, whereby patients with EDS have skin hyperextensibility secondary to the alignment of dermal collagen bundles in the line of force.^8^ This allows for an initial extension of skin when pinched, but stops any further skin deformity when considerable additional force is applied. Furthermore, a correlation between skin hyperextensibility and increasing joint hypermobility has been shown, which may be variable depending on ethnicity.^8^ For cEDS patients, skin hyperextensibility has been shown to be greater than in healthy controls, with the skin lifting more than 1.5 cm from the dorsal surface of the non‐dominant distal forearm or dorsum of the hand (Figure 1a).^1^ Skin hyperextensibility is most easily assessed over the elbows, lateral neck and knees and extensibility of 3 cm or more at these sites is suggestive of cEDS.^9^ Lesser degrees of extensibility affect the other EDS subtypes.

### 3.2 Atrophic scarring and bruising

Atrophic scarring is defined as scars from linear traumatic lacerations or a single‐surgery that are unusually shallow (i.e. thin and sunken) and/or wider than the original wound due to impaired repair and subsequent dermal hypotrophy.^3^ Fragile skin with marked atrophic scarring is common over the forehead, chin, shins and extensor surfaces (Figure 1b). Mild trauma to the skin often results in avulsion style lacerations and easy bruising.^9^ Scars located over the limbs often have a ‘horseshoe’ or ‘fishmouth’ appearance. Haemosiderin deposition within scars is common in cEDS and reflects chronic capillary leakage of red blood cells from chronic microtrauma similar to that affecting the skin in elderly individuals.^9^

Premature bruising after minimal trauma is first observed in cEDS when children begin crawling/walking and requires further assessment of joint hypermobility, scarring and skin hyperextensibility.^10^ Bruising often occurs spontaneously, but is common at trauma‐prone sites such as the anterior aspects of the lower limbs and the extensor aspects of the forearms. The differential diagnoses of paediatric bruising include clotting disorders and non‐accidental injury, necessitating careful evaluation.^11^

### 3.3 Molluscoid pseudotumours and subcutaneous spheroids

Molluscoid pseudotumours are fleshy, fibrotic nodules that can be up to 3 cm in diameter seen with scars on pressure points, such as the elbows and knees.^6^ They follow repeated trauma and haemorrhages. Subcutaneous spheroids are hard pea‐sized or smaller mobile, palpable lesions over the ulna and tibia due to calcification of fat lobules after trauma, which are also visible on radiographs.^9^

### 3.4 Other cutaneous features

Premature skin softening can occur and increases with age. Cutaneous texture is often described as doughy or velvety as detectable by palpation of the dermis over the forearms.^6^ Epicanthic folds are common in some cEDS families, but are not specific to cEDS and are also seen in many other genetic disorders including Trisomy 21, Turner, Noonan, Williams and Rubinstein‐Taybi syndromes and phenylketonuria.^12^ Blepharochalasis and infraorbital creases are ‘soft signs’ that may help guide a clinician towards a possible diagnosis of cEDS.^13^ Excess eyelid skin and prematurely aged skin over the face, hands and feet are also described.^14^

## 4 VASCULAR EHLERS DANLOS SYNDROME

Vascular EDS (vEDS) is caused by heterozygous pathogenic variants in *COL3A1*, which result in defective or reduced secretion of collagen III by skin fibroblasts.^15^ Type III collagen is widely distributed in the dermis of the skin, pleuro‐peritoneal linings, pelvic ligaments, intestinal tract (including the gingiva), as well as in venous and arterial walls, strongly reflecting the areas affected in vEDS.^15^ It is particularly important to recognize this subtype due to the high risk of arterial, as well as colonic rupture with some cases documented prior to adolescence.^16^ During pregnancy uterine rupture can occur with associated mortality.^12^ There may be no family history as de novo mutations occur in up to 50% of cases.^17^

### 4.1 Translucent skin

**FIGURE 2:**
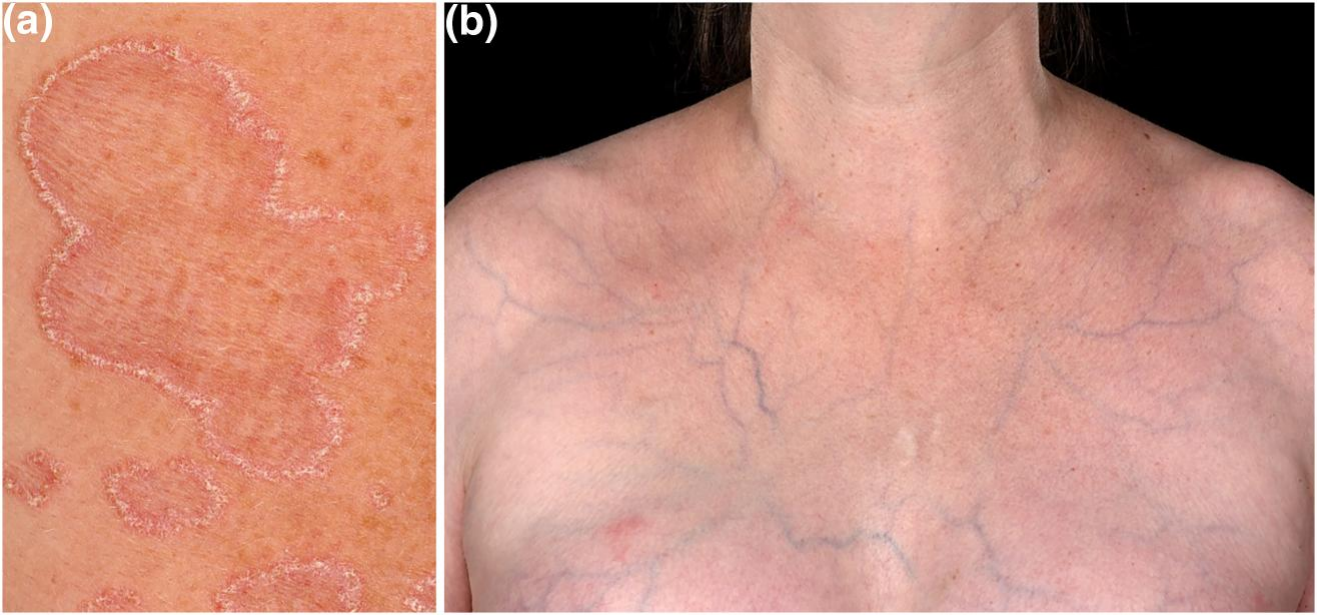
Cutaneous features of vascular Ehlers‐Danlos syndrome including (a) elastosis perforans serpiginosa, which presents with erythematous keratotic papules in serpiginous or arcuate configurations and atrophic centres and, (b) thin translucent skin with visible veins and spontaneous ecchymoses. Images courtesy of Addenbrooke's Hospital, Cambridge University Hospitals NHS Foundation Trust, United Kingdom

Whilst vEDS patients do not usually have significant skin hyperextensibility, most commonly show skin translucency due to a decrease of collagen as a consequence of reduced type III dermal collagen, secondary to a *COL3A1* mutation.^18^ Dermal thinning is most evident over the dorsum of the hands, upper chest and shoulders (Figure 2a). When thinning occurs over the face, hands and feet, with fine wrinkling, it is referred to as acrogeria.^19^ Wrinkling is a consequence of a lack of skin turgor, secondary to a lack of collagen, which is needed to hold water within the skin. Acrogeria is also a feature seen in some premature ageing syndromes and Loeys‐Dietz syndrome, and to a lesser degree in Periodontal EDS (pEDS) and clEDS.^19^

### 4.2 Atrophic scarring and bruising

Atrophic scars, especially of the knees and shins are also common due to skin fragility, but scarring is generally narrower than in cEDS. Bruising is a cutaneous hallmark of this subtype. Patients also typically bruise more easily and widely than in other EDS subtypes (except pEDS) with minimal trauma, in unusual sites and occasional large haematoma formation are encountered.

### 4.3 Other cutaneous features

Elastosis perforans serpiginosa is characterized by the transepidermal elimination of abnormal elastic fibres through the skin.^20^ Typically, erythematous keratotic papules develop in serpiginous or arcuate configurations leaving slightly atrophic centres (Figure 2a). Whilst not specific to EDS it is seen more frequently in vEDS and other connective tissue disorders.^21^ Features that may be present include gingival recession, and early onset varicose veins.^3^ Diffuse hair thinning appears to also be more common in vEDS although the mechanism is not known. A definite facial phenotype is associated with some vEDS patients, although it may be subtle. Lobeless pinnae, deep‐set eyes, a thin face lacking in fat volume, pinched nose and thin upper lip also are suggestive of vEDS.^15^

## 5 PERIODONTAL EHLERS DANLOS SYNDROMES

Periodontal EDS derives its name from the prominence of oral and mucosal features, namely severe and intractable periodontitis with onset in childhood or adolescence.^3^ This leads to the loss of keratinized gingiva and gingival recession.^3^, ^13^ It appears to be caused by heterozygous gain‐of‐function mutations in *C1R* or *C1S*, which encode the first components of the classical complement pathway.^22^ The mechanism of periodontal thinning is presently unclear, but increased collagen degradation mediated by complement changes is possible.

### 5.1 Pretibial plaques

**FIGURE 3:**
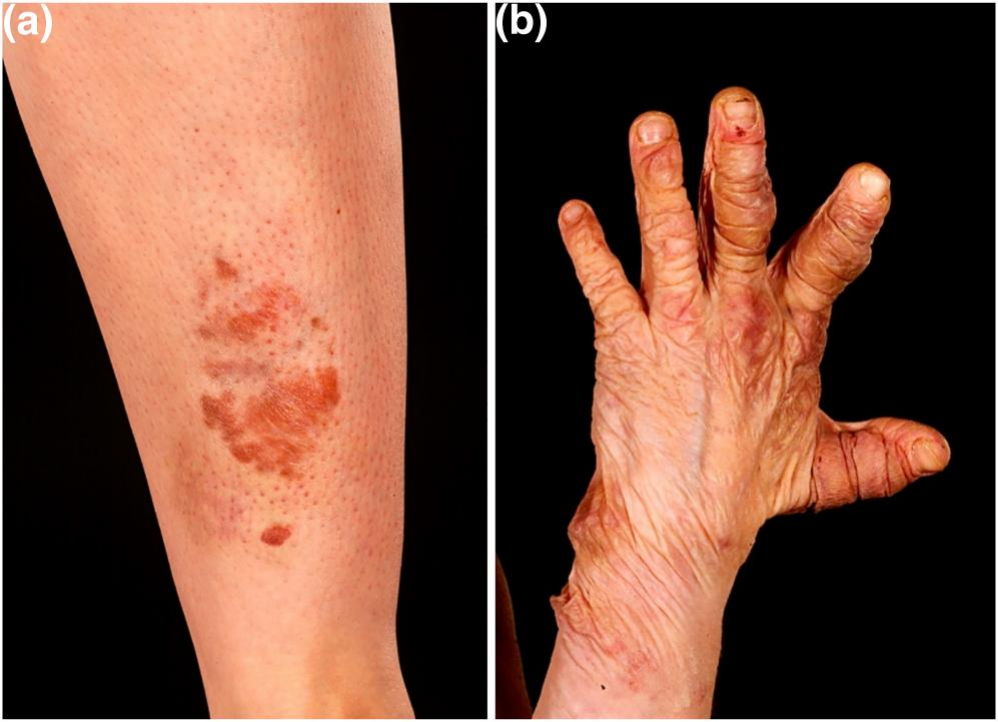
(a) Pretibial plaques seen in periodontal Ehlers‐Danlos syndrome and, (b) sagging skin and redundancy observed in dermatosparaxis Ehlers‐Danlos syndrome. Images courtesy of Addenbrooke's Hospital, Cambridge University Hospitals NHS Foundation Trust, United Kingdom

Pigmented pretibial shin plaques are suggestive, but not specific for pEDS and are common in vEDS and cEDS (Figure 3a). Histologically there are fibrosis and haemosiderin deposits and overlap and resemblance to necrobiosis lipoidica. Individuals with pEDS also exhibit skin hyperextensibility and fragility with atrophic scars and may also have prominent vasculature, and/or acrogeria.^22^

## 6 DERMATOSPARAXIS EHLERS DANLOS SYNDROME

In dermatosparaxis EDS (dEDS), the skin is extremely and prematurely fragile with congenital or postnatal tears and sagging around the wrists and ankles (Figure 3b).^23^ Palmar wrinkling, atrophic scarring, marked bruising with subcutaneous haematomas, soft doughy skin and postnatal growth retardation with short limbs, hands and feet may also be present.^3^ Craniofacial features can occur at birth or later in childhood and include prominent eyes with oedematous eyelids, blepharochalasis, epicanthal folds, large fontanelles and delayed closure.^3^

## 7 HYPERMOBILE EHLERS DANLOS SYNDROME

Hypermobile EDS (hEDS), which overlaps with hypermobility spectrum disorders relies on a clinical diagnosis as no consistent or common genetic cause has yet been identified.^24^ Furthermore, the current diagnostic criteria for hEDS were designed to identify patients with a consistent phenotype for gene discovery.^3^ Cutaneous alterations are much milder in all respects than those in cEDS. The diagnostic criteria include mild skin hyperextensibility, soft skin, unexplained striae (such as striae distensae or rubrae at the back, groin, thighs, breasts and/or abdomen) and mild atrophic scarring involving at least two sites and without the formation of truly papyraceous and/or haemosiderin scars seen in cEDS.^3^ True skin fragility, such as the propensity to have an open wound due to trivial trauma, is not a typical feature of hEDS. Significant joint hypermobility and associated complications such as chronic pain, and dislocations are key.^25^ Bilateral piezogenic papules affect the medial and lateral aspects of the ankles of the foot and represent herniation of underlying soft tissue but are not specific to hEDS. Additional features such as easy bruising, haematomas, blue sclerae and delayed wound healing were also noted in a cross‐sectional review of joint hypermobility syndrome/hEDS.^26^ The differential diagnosis of hEDS includes clEDS and Cardiac‐valvular EDS (cvEDS).

## 8 CLASSICAL‐LIKE EHLERS DANLOS SYNDROME

Autosomal recessive *TNXB* mutations cause classical‐like EDS (clEDS), with cutaneous features resembling cEDS with hyperextensibility, easy bruising and/or slow wound healing.^23^ It has also been noted that some clEDS patients exhibit oedema in the legs in the absence of cardiac failure.^3^, ^27^ The critical difference between clEDS compared to cEDS is the absence of atrophic or atypical scars. Acrogeric skin thinning, pelvic prolapse and various foot deformities including pes planus and hallux valgus are non‐specific findings.^3^ The latter can also be seen in vascular and arthrochalasia subtypes.

## 9 RARER EHLERS DANLOS SYNDROMES SUBTYPES WITH CUTANEOUS FEATURES

Cardiac‐valvular EDS is an important subtype and is accompanied by aortic and/or mitral valve pathology.^23^ Cutaneous features resemble cEDS with hyperextensibility, atrophic scarring and bruising. Variants affecting the alpha 2 chains of type I collagen result in cardiac valve pathology.^28^ In other cases, the clinical phenotype of these variants cause in severe osteogenesis imperfecta type II/III.^29^ Given the possible clinical overlap between cEDS and cvEDS, a baseline echocardiogram should be considered for cEDS patients to screen for cardiac pathology that may indicate a missed diagnosis of cvEDS. The subtype may also present with pectus excavatum, inguinal hernias and foot deformities.^3^

Arthrochalasia EDS can present with skin hyperextensibility, but atrophic scarring and bruising have previously been listed as minor non‐specific features. Congenital bilateral hip dislocation with generalized joint hypermobility, hypotonia, kyphoscoliosis and mild osteopenia are present. Furthermore, a crisscross patterning of the palms and soles has also been noted in some cases.^23^ In musculocontractural EDS congenital adduction‐flexion contractures are found, as well as talipes equinovarus with skin hyperextensibility, bruising, fragility with atrophic scars and increased palmar wrinkling.^3^ Furthermore, peculiar tapered, slender fingers, hyperalgesia to pressure and fistula formation due to recurrent subcutaneous infections has been noted in this subtype.^23^

In kyphoscoliotic EDS the major clinical features of congenital muscle hypotonia and kyphoscoliosis present in infancy or childhood alongside generalized hypermobility.^1^ Cutaneous signs are relatively non‐specific but include skin hyperextensibility, easy bruising and keratosis pilaris. Features specific to heterozygous pathogenic variants in *PLOD1* include fragility of skin, blue sclera and ocular issues (ocular fragility, high myopia, glaucoma and microcorneas).^30^ Congenital hearing impairment and follicular hyperkeratosis are reported with heterozygous pathogenic variants in *FKBP14*.^3^, ^31^ Other cutaneous features described with pathogenic variants of *FKBP14* include molluscoid pseudotumours, multiple isolated comedones and umbilical skin redundancy.^32^ Urinalysis by high performance liquid chromatography can show an increased dexoypyridinoline to pyridinoline ratio, specific for *PLOD1* mutations, which is not present with *FKBP14* mutations.^3^

Brittle cornea syndrome causes a thin cornea, keratoconus and keratoglobus with myopia and deafness, whilst limited skin signs include soft, velvety and translucent skin, without atrophic scarring.^3^, ^23^ Myopathic EDS displays congenital muscle hypotonia and joint contractures are major features along with hypermobile small joints, whilst skin changes can include softness and atrophic scarring.^1^ Spondylodysplastic EDS presents with progressive short stature in childhood, with hypotonia and bowing of limbs.^3^ Skin hyperextensibility and softness can be present, with thin and translucent skin.

Ehlers Danlos syndromes cases caused by *AEBP1* autosomal recessive pathogenic variants are limited but are considered a classical‐like EDS (clEDS2) due to the significant clinical overlap with cEDS, including skin hyperextensibility, atrophic scarring and generalized joint hypermobility. The mode of inheritance and early‐onset osteopenia are helpful points of differentiation. Specific cutaneous features per subtype can be found in Supplementary Information (see Fig. S1).

## 10 HISTOLOGICAL FEATURES IN DIFFERENT EHLERS DANLOS SYNDROMES SUBTYPES

In addition to cutaneous findings, both light and transmission electron microscopy (TEM) of skin biopsy samples are helpful in some subtypes,^33^ although not diagnostic on their own, as these findings are largely qualitative (Table 2).^34^ A recent analysis of TEM in 24 patients with a definitive diagnosis of monogenic EDS noted that 17 (71%) had an abnormal biopsy report.^35^ They also noted that no TEM findings were specifically associated with any EDS subtype, although collagen flowers were present in most patients with a genetically confirmed diagnosis of cEDS.^35^ These results also need to be interpreted with patient age and current treatment in mind, as a thin dermis can also result from photoageing of skin or from prolonged use of topical corticosteroids.^36^ If TEM is not easily available, preliminary light microscopy is helpful when stained for collagen and elastin, particularly for vEDS and cEDS.

**TABLE 2:** Light and electron microscopy findings reported in Ehlers Danlos syndromes (EDS) subtypes^a^

EDS subtype	Light microscopy	Electron microscopy
Classical	Suggestive signs are loose and dispersed dermal collagen and variable bundle size (transmission electron microscopy).	Collagen rosettes representing aberrant collagen fibrils with regular dermal distribution are common and highly characteristic.
Vascular	Reduced collagen to elastin ratio seen with collagen depletion and elastic prominence. A thin dermis is strongly suggestive.	Variability of collagen fibril diameter in skin, arterial and intestinal samples.
Periodontal	Decrease collagen to elastin ratios (resembling vascular EDS).	Variability of collagen fibril diameter similar to vascular EDS.
Dermatosparaxis	Moderately disorganized deep dermal collagen fibres may be found.	Aberrant or hieroglyphic patterns of collagen fibrils reflect pathologic procollagen cleaving are specific.
Arthrochalasia	Loose and dispersed dermal collagen with rare bundles.	Lower fibril density and occasional cauliflower deformations of the fibrils.

Abbreviation: EDS, Ehlers‐Danlos syndrome.

^a^ Please refer to the Supplementary File for references used in Table 2.

This review highlights those skin signs helpful to a potential EDS diagnosis in some EDS subtypes, particularly cEDS, vEDS, pEDS, dEDS and clEDS2 subtypes. In patients with signs of cEDS such as skin hyperextensibility, fragility and atrophic scarring there are other differentials to consider including clEDS, *AEBP1*‐related EDS and cvEDS. Vascular EDS patients typically have translucent skin, though this can be seen in other subtypes and may lack large joint hypermobility and may instead have hypermobility of smaller joints. Periodontal EDS patients typically have severe premature and progressive periodontitis presenting in early childhood and pretibial plaques may be seen but are not pathognomonic as these can overlap with cEDS and vEDS. Dermatosparaxis EDS patients have extreme, premature skin fragility with resultant redundant, skin laxity. These subtypes may present in the dermatology clinic and in the case of vEDS, if unrecognized can have life‐threatening complications. Clinicians can identify signs suggestive of these EDS subtypes on skin examination, but features can overlap between subtypes. Skin biopsies for light and electron microscopy provide additional evidence to further support the need for formal DNA analysis. Genotyping is becoming increasingly accessible and is an important addition to the dermatologist's tool kit. This is especially true for vEDS, where confirmation of diagnosis allows for predictive testing of other at‐risk family members, affording appropriate early intervention, in the prevention of the severe internal manifestations of the disorder. For all subtypes where the genetic basis is known, reproductive options such as pre‐implantation genetic testing may be facilitated, with appropriate counselling.

## AUTHOR CONTRIBUTIONS

**Nastasha Stembridge**: Data curation (Equal); Formal analysis (Lead); Investigation (Lead); Writing – original draft (Equal); Writing – review & editing (Supporting). **Brent J. Doolan**: Formal analysis (Equal); Resources (Equal); Writing – review & editing (Lead). **Mark E. Lavallee**: Writing – review & editing (Supporting). **Ingrid Hausser**: Writing – review & editing (Supporting). **F. Michael Pope**: Writing – review & editing (Supporting). **Suranjith L. Seneviratne**: Writing – review & editing (Supporting). **Ingrid M. Winship**: Writing – review & editing (Supporting). **Nigel P. Burrows**: Conceptualization (Equal); Methodology (Lead); Supervision (Lead); Writing – original draft (Equal); Writing – review & editing (Supporting).

## CONFLICT OF INTEREST

All authors of this manuscript certify that they have no affiliations with or involvement in any organization of entity with any financial interest or other equity interest or non‐financial interest in the materials discussed in this manuscript.

## Supporting information

Supplementary Information S1

## Data Availability

Data sharing is not applicable to this article as no new data were created or analyzed in this study.
