# Supplementary material for: The role of cutaneous manifestations in the diagnosis of the Ehlers‐Danlos syndromes
Source: Skin Health Dis. 2022 Jul 15;3(1):e140. doi: 10.1002/ski2.140 (PMC9892481; doi:10.1002/ski2.140)
Supplement: Supplementary file 1 — Supplementary Information S1 [file SKI2-3-e140-s001.docx]

**The role of cutaneous manifestations in the diagnosis of the Ehlers-Danlos Syndromes**

Stembridge N, Doolan BJ, Lavallee M, Hausser I, Pope FM, Seneviratne SL, Winship IM, Burrows NP

**SUPPLEMENTARY INFORMATION:**

**Figure S1:** Outline of cutaneous features and their associated Ehlers-Danlos syndrome subtype


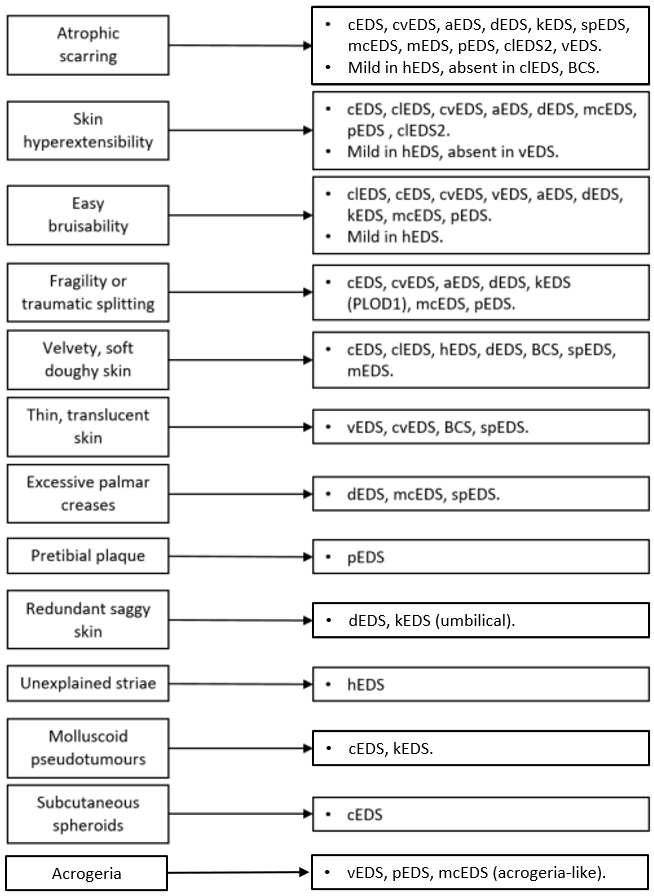


**References for Table 1 and Table 2:**

- Malfait F, Francomano C, Byers P *et al.* The 2017 international classification of the Ehlers-Danlos syndromes. *Am J Med Genet C Semin Med Genet* 2017;**175**:8-26.
- Bowen JM, Sobey GJ, Burrows NP *et al.* Ehlers-Danlos syndrome, classical type. *Am J Med Genet C Semin Med Genet* 2017;**175**:27-39.
- Angwin C, Ghali N, Baker D et al Electron microscopy in the diagnosis of Ehlers-Danlos syndromes: correlation with clinical and genetic investigations. *Br J Dermatol*. 2020;**182**(3):698-707.
- de Almeida HL, Jr., Bicca E, Rocha NM *et al.* Light and electron microscopy of classical Ehlers-Danlos syndrome. *Am J Dermatopathol* 2013;**35**:102-5.
- Van Damme, T., Colige, A., Syx, D. et al. Expanding the clinical and mutational spectrum of the Ehlers–Danlos syndrome, dermatosparaxis type. *Genet Med* 2016;**18**:882–891.
- Proske S, Hartschuh W, Enk A, Hausser I. Ehlers Danlos Syndrom -- 20 Jahre Erfahrungen in Diagnostik und Klassifikation an der Universitäts-Hautklinik Heidelberg. *J Dtsch Dermatol Ges*. 2006;**4**:308-18.
- Klaassens M, Reinstein E, Hilhorst-Hofstee Y *et al.* Ehlers-Danlos arthrochalasia type (VIIA-B)-expanding the phenotype: from prenatal life through adulthood. *Clin Genet*. 2012;**82**:121-130.
